# An unusual case of vaginal myiasis

**DOI:** 10.1099/jmmcr.0.005060

**Published:** 2016-12-16

**Authors:** Hannah Soulsby, Brian L. Jones, Michael Coyne, Claire L. Alexander

**Affiliations:** ^1^​ Clinical Microbiology, Glasgow Royal Infirmary, Glasgow, Scotland; ^2^​ Scottish Parasite Diagnostic and Reference Laboratory, Glasgow, Scotland, UK

**Keywords:** Myiasis, vagina, larvae, blow fly

## Abstract

**Introduction:**

Myiasis, a term used to describe the infestation of a live animal by fly larvae, is rarely reported in human subjects. The adult fly lays its eggs on living tissue that progresses to become larvae that feed on living tissue having gone through three developmental stages known as the first, second and third instar. The larvae become pupae before finally developing into adults.

**Case presentation:**

We describe an unusual case of a 79-year-old female who collapsed in her garden and lay there for several days before presenting to her local hospital Accident and Emergency department with an infestation of larvae in her vagina labia, identified as those from the *Protophormia* species northern blowfly. After complete removal of the larvae using tweezers followed by cleansing of the affected area and a course of antibiotics, the patient’s condition improved. A follow-up review by the local gynaecology team revealed no evidence of further infestation.

**Conclusion:**

It is our understanding that this is the first highly unusual case of a blowfly larvae infestation to be reported in a human within the UK.

## Introduction

Insects of the order Dipteran account for one of the largest orders of human and veterinary importance. There exists over 100 000 described species with complete metamorphosis occurring in the four stages: egg, larva, pupa and adult. Female adults deposit their eggs into a nutrient-rich substrate, which can include animal or human tissue, and after hatching, the larvae continue feeding throughout three stages termed the first, second and third instar. Each instar transition is marked by a molt that ends with the third instar larvae developing into pupa, from which adult blowflies emerge after 6–8 days. The adults are attracted to the natural moist body openings, open wounds or pooled blood of the animal or human host. The larvae, also termed maggots, feed on living or decaying material, food sources or body fluids. Infestation of living tissue by fly larvae is described as myiasis, a term coined in 1965 that can be classified as bloodsucking, cutaneous, wound or, in this case, cavitary (Francesconi *et al.*, 2012). Myiasis by Dipteran insects has been reported in livestock within Scotland and the rest of the UK causing ‘sheep strike’. This involves maggots invading exposed, often wounded tissue, and feeding off of blood from the host that may ultimately result in death through extensive tissue damage. Another form is cavitary myiasis that involves invading body cavities such as the nose, mouth, anus or vagina. Both cutaneous and cavitary myiasis are the most common forms encountered in humans. Myiasis larvae are legless and have soft bodies with respiration occurring via structures known as spiracles that are extremely useful features to assist with laboratory identification of the larvae. Larvae sent to specialist reference laboratories for identification are commonly staged as third instar. We present a highly unusual case of myiasis by *Protophormia* species, which describes the infestation of an elderly patient’s labia by the larvae from a common fly in the United Kingdom, known as the northern blowfly, blue bottle fly or blue-arsed fly.

## Case report

A 79-year-old lady with a background of multiple spinal fractures resulting in chronic back pain, poor mobility and falls, osteoporosis and mild cognitive impairment was admitted to the Queen Elizabeth University Hospital, Glasgow, during August 2015. She was found unconscious in the garden by a neighbour and the paramedics were called to the scene. She was unable to mobilise and had been positioned in the one location unnoticed for a minimum of 2 days. On admission to the local hospital’s Accident and Emergency (A&E) department, the patient was confused, dehydrated, septic and sunburnt. She had abrasions over her thoracic and sacral area with cellulitis. When her trousers were removed, it was noted that a fly flew out. Further investigations revealed maggots embedded in her vagina labia. A fracture to the right neck of her femur, rhabdomyolysis and a urinary tract infection were also noted.

### Investigations

The maggots, six in total, were removed from the labia using tweezers and sent to the Scottish Parasite Diagnostic and Reference Laboratory (SPDRL), Glasgow Royal Infirmary. On arrival at the SPDRL, the live maggots were killed by immersing in boiling water for 60 s before being submerged in 90 % ethanol. The final segment containing the posterior spiracles of the segmented body was dissected using a sterile blade before being submerged into 5 % potassium hydroxide. This was gently heated to 100 °C for 5 min. Once cooled, the segment was rinsed in distilled water then transferred to a glass slide and examined under both the dissecting microscope and a light microscope using ×10 magnification.

### Diagnosis

**Fig. 1.**
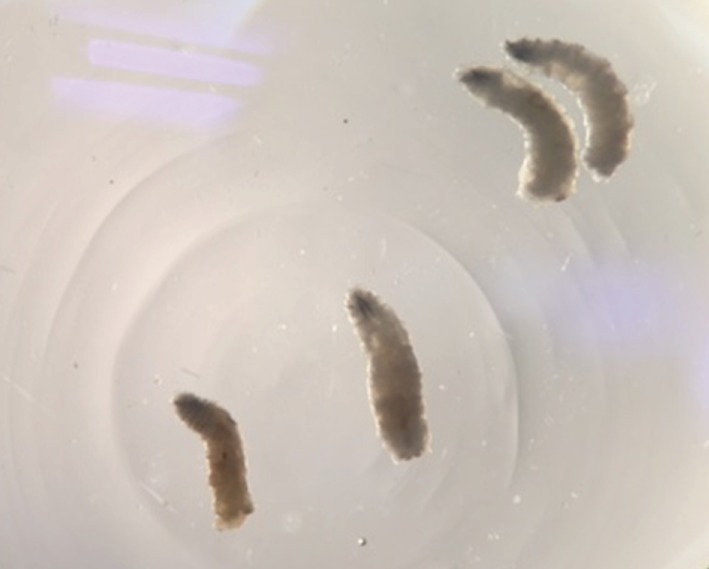
Second instar *Protophormia* species larvae removed from the patient’s labia.

**Fig. 2.**
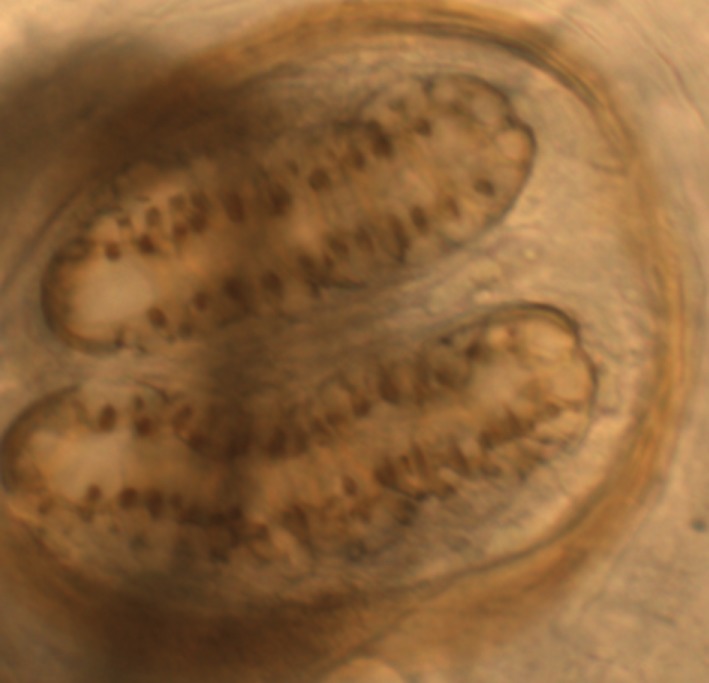
Posterior spiracle formation of the second instar larvae.

The second instar larvae (Fig. 1) showing the spiracles (Fig. 2) were identified as those from the *Protophormia* species northern blowfly of the family *Calliphoridae* (ITIS, 2015).

### Treatment

The patient’s condition improved after complete removal of the larvae followed by cleansing of the infected area, intravenous fluids and antibiotics. She remained well enough to go to theatre for a dynamic hip screw 10 days post admission.

### Outcome and follow-Up

The patient was reviewed by the Gynaecology Team 4 days later, and there was no evidence of an ongoing vaginal infestation. The patient was transferred to a local rehabilitation centre and made good progress. She was discharged home late November 2015.

## Discussion

This report describes a highly unusual case, where it is most likely that the adult fly/flies opportunistically took advantage of an opening in the skin, e.g. scratch wound, or other appropriate conditions in the labia to support the life cycle of the fly (Burgess, 2003). When the eggs from this fly are laid, they hatch into maggots within 12–24 h. The first instar larvae develop by approximately 1.8 days; the second instar, by 2.5 days; and the third instar, around 4–6 days (Burgess, 2003). Based on the last known sightings from neighbours, the length of time that the patient was thought to have spent lying in the garden was approximately 2 days; therefore, the adult fly observed in A&E was unlikely to have gone through this cycle of development whilst embedded in the patient. A more likely explanation was that the adult fly was in the process of laying eggs and starting the cycle when it became disturbed on removal of clothing. The larvae identified from the labia were the second instar, which is the stage of development reached by around 2.5 days, and this fits with the length of time the patient was likely to have been in the garden. The first instar larva obtained its nutrients originally from the body fluids surrounding the labia that is promoting its growth, whilst the second instar stage with the more developed mouthparts began to digest the labia tissue.

There is limited information in the literature describing human cases of myiasis. A review of wound myiasis in urban United States estimated that there may be as many as 7000 cases per year, but many cases are likely to go unreported due to cultural and social reasons (Sherman, 2000). Risk factors for myiasis include debilitation, blood or body odor, neglect of personal hygiene, alcoholism and the summer season (Greenberg, 1984). Three of these factors were present in this case, and additionally, the increased outdoor exposure was likely to have contributed by increasing the potential exposure to flies. Other factors known to increase risk of myiasis include diabetes or peripheral vascular disease, which are associated with chronic wounds (Sherman, 2000).

Management of a patient with blowfly myiasis involves cleansing of the wounds and tetanus updates (Sherman, 2000). Occasionally debridement is required if the maggots make there way into tissue (Burgess & Spraggs, 1992). Blood cultures are recommended to ensure that there is no secondary sepsis with antibiotics being prescribed if there is evidence of active bacterial infection (Sherman, 2000). Cases have also been treated with ivermectin, though generally when patients have orbital myiasis or severe invasive infection (Osorio *et al.*, 2006; De Tarso *et al.*, 2004; Costa *et al.*, 2005).

The blowfly is a very important species in forensic entomology where the stages of insect development can be analysed to determine the time elapsed between a person's death and the discovery of the body (Catts & Goff, 1992). This is defined as the post-mortem interval (PMI). Although the human body can be infested with a variety of different insects during the years it may take to fully decompose, blow flies are usually the first insects to colonise a body, frequently within minutes after death (Catts, 1992). They are attracted to the natural moist body openings, open wounds or pooled blood of the victim. Maggot age, internal contents and development can give a date of death accurate to a day or less (Catts, 1992). Several factors affecting PMI estimates include the weather conditions, metabolic heat generated by the mass of maggots in the corpse and the presence of clothing or other insect species (Catts & Goff, 1992; Catts, 1992). Blowfly larvae isolated from the body can be used to test the corpse for the presence of poisons and drugs with some drugs speeding up whilst others slow down development of the insect (Catts & Goff, 1992; Catts, 1992). Mercury has an adverse effect on the growth of maggots whilst cocaine has been shown to accelerate growth (Nuorteva & Nuorteva, 1982; Goff *et al.*, 1989). Toxins such as phenobarbitone can be detected in maggots that have developed on a corpse (Kintz *et al.*, 1990).

Historically, due to their flesh-eating ability, maggots have played a role in wound management. Before antibiotics, larval therapy was widespread in Europe with batches of 200–600 maggots being applied to wounds that were then covered for 3–5 days. *Protophormia* species was one of the species that was used at this time. After 1940, few clinicians chose to use maggots, in favour of antibiotics (Burgess, 2003). However, with the development of antimicrobial resistance, it may be appropriate for the blowfly maggots to be put to use again, especially with developments in wound dressings that have reduced the rates of previous complications (Sherman, 2009). It can be a particularly cost effective therapy in diabetic foot infections (Sherman, 2003, 2009; Tian *et al.*, 2013) with potential savings in the UK estimated at £50 million annually (Thomas, 2006).
